# Communicating *PNPLA3* genetic risk status for NAFLD among Mexican-origin men

**DOI:** 10.3389/fpubh.2022.1090101

**Published:** 2023-01-04

**Authors:** Edgar A. Villavicencio, Adriana Maldonado, Rebecca M. Crocker, Yue Guan, Chris Stallman, David O. Garcia

**Affiliations:** ^1^Department of Health Promotion Sciences, Mel and Enid Zuckerman College of Public Health, University of Arizona, Tucson, AZ, United States; ^2^Center for Border Health Disparities, Health Sciences, University of Arizona, Tucson, AZ, United States; ^3^Rollins School of Public Health, Emory University, Atlanta, GA, United States; ^4^Genetic Counseling Graduate Program, University of Arizona, Tucson, AZ, United States

**Keywords:** NAFLD, genetic risk, *PNPLA3*, Mexican-origin, men's health

## Abstract

**Introduction:**

The burden of non-alcoholic fatty liver disease (NAFLD) continues to disproportionately impact under-resourced communities in the U.S., particularly Mexican-origin populations. Genetic polymorphisms such as the rs738409 C/G variant in patatin-like phospholipase domain-containing 3 (*PNPLA3*) have been associated with higher prevalence of and progression along the NAFLD spectrum. This qualitative study conducted in the U.S. Southwest aimed to assess Mexican-origin men's experience receiving genetic testing for *PNPLA3* risk carrier status.

**Methods:**

Semi-structured interviews were conducted with 17 Mexican-origin men whose NAFLD status and genetic predisposition were determined as part of a previous cross-sectional study. The interview guide included questions exploring participants' insights on how genetic risk status was delivered, how the information influenced their motivation for lifestyle modification to reduce NAFLD risk, and any knowledge sharing that occurred with family members after learning of their *PNPLA3* risk status. Interviews were conducted and audio recorded in English (*n* = 6) and Spanish (*n* = 11) and uploaded into NVivo software for data analysis and interpretation. Guided by the Health Belief Model, a thematic analysis approach was used to identify primary themes.

**Results:**

Results highlighted men's preference for receiving this type of genetic risk information through a letter sent to their homes. General comprehension of *PNPLA3* risk status was deemed high and most men stated sharing their genetic predisposition to NAFLD with their immediate family members. Participants also indicated that family and awareness of this genetic risk acted as primary motivators for implementing behavior changes (e.g., diet, physical activity) toward the prevention of more severe liver conditions.

**Discussion:**

Findings from this qualitative study suggest the feasibility of communicating genetic risk for NAFLD among Mexican-origin men. Future strategies for the dissemination of genetic risk results among Mexican-origin individuals should consider familial and cultural appropriate strategies.

## Introduction

As the most common liver disease in United States (U.S.), non-alcoholic fatty liver disease (NAFLD) affects ~25% of the U.S. population (1). NAFLD is a multifaceted disease resulting from a complex interplay of genetic, environmental, metabolic and microbial factors disproportionately impacting Mexican-origin adults (2). Compared to non-Hispanic Blacks (21.6%), non-Hispanic Whites (30.6%), and other Hispanic subpopulations (27.6%), NAFLD rates are the highest among Mexican-origin adults (42.8%) (3). The differences among racial and ethnic groups are partially explained by obesity status, sex differences (higher rates among men), and genetic polymorphisms such as the rs738409 C/G variant in patatin-like phospholipase domain-containing 3 (*PNPLA3*) (4).

The single nucleotide polymorphism (SNP) represents an Ile148Met substitution (C > G) in the *PNPLA3* gene (5). Specifically, differences in the C and G variants are due to changes in an amino acid from isoleucine (I) to methionine (M) at the position 148 (I1e148 Met) of the protein, which is associated with high frequency of fat cells in the liver (6). *PNPLA3* is associated with an increased risk for NAFLD across the full spectrum of the disease including age of diagnosis, steatosis, fibrosis, and hepatocellular carcinoma (HCC) (7, 8). Romeo et al. (9) were among the first to show individuals carrying two copies of the risk allele displayed levels of hepatic fat content that was greater than two-fold higher than non-carriers. In addition, this study found that Hispanic descent individuals had a higher frequency (49%) of *PNPLA3* compared to European Americans (23%) and African Americans (17%) (9). More recently, Martínez et al. (10) found Mexicans living in Mexico carrying two copies of the risk allele had 3.8 times higher risk of having NASH and 2.3 times higher risk of fibrosis. This is concerning as estimates from Mexican-origin populations suggest the frequency of the G risk allele is up to 77% (10, 11). Despite the significant genetic basis for *PNPLA3* testing for Mexican-origin adults, there are currently no recommendations for routine testing of this genetic variant in screening or clinical care of the disease. However, this may change as more evidence becomes available (12), making the examination of appropriate and effective genetic risk communication strategies a critical step toward the widespread communication of *PNPLA3* susceptibility.

While the importance of genetic testing has recently emerged within the context of personalized medicine, there have been limited studies on genetic testing strategies for Mexican-origin adults and no studies to date specifically for men (13–16). However, low levels of knowledge and awareness about genetic testing have previously been reported (16), and prior findings demonstrate that Hispanics may not understand risk within the context of genetics due to limited genetic literacy defined as the ability to obtain, process, understand, and use genetic information (17, 18). This may be due in part to limited health care access and health information faced by many Hispanics in the U.S. (19). Lower awareness and exposure to genetic testing is also most prominent among Hispanics with lower levels of acculturation (20, 21). In addition, barriers to the adoption of genetic testing for Hispanics have been identified such as concerns about misuse of genetic information, personal utility (behavioral and lifestyle implications), and adverse emotional responses (fear of receiving an alarming result) (16). Practical barriers identified include finances (related to the costs of genetic testing and necessary follow-up medical care), a lack of access and knowledge about where to get a genetic test, a lack of a family history of disease, and difficulties related to language proficiency and educational level (16). Despite these barriers, existing results indicate that Hispanics have an interest in and favorable attitudes toward genetic testing (16, 22).

Efforts to promote genomic risk communication is often limited to single-gene disorders (e.g., BRCA1 or BRCA2 for breast and ovarian cancer prevention) (23). Further, screening efforts focused on genomic risks presume that knowledge of risk levels should promote risk-appropriate uptake of health prevention behaviors. However, understanding complex and unfamiliar concepts associated with genomic risk can be challenging. Given the genetic heritability of NAFLD and the fact that lifestyle modification is the cornerstone of treatment for risk reduction (24), efforts to improve knowledge and awareness of *PNPLA3* risk status are important to inform therapeutic lifestyle strategies. This qualitative study conducted in the U.S. Southwest aimed to assess Mexican-origin men's experience receiving genetic testing for *PNPLA3* risk carrier status. The interview guide included questions exploring participants' insights on how genetic risk status was delivered, how the information influenced their motivation for lifestyle modification to reduce NAFLD risk, and any knowledge sharing that occurred with family members after learning of their *PNPLA3* risk status.

## Methods

### Design, study sample and recruitment

**Figure 1:**
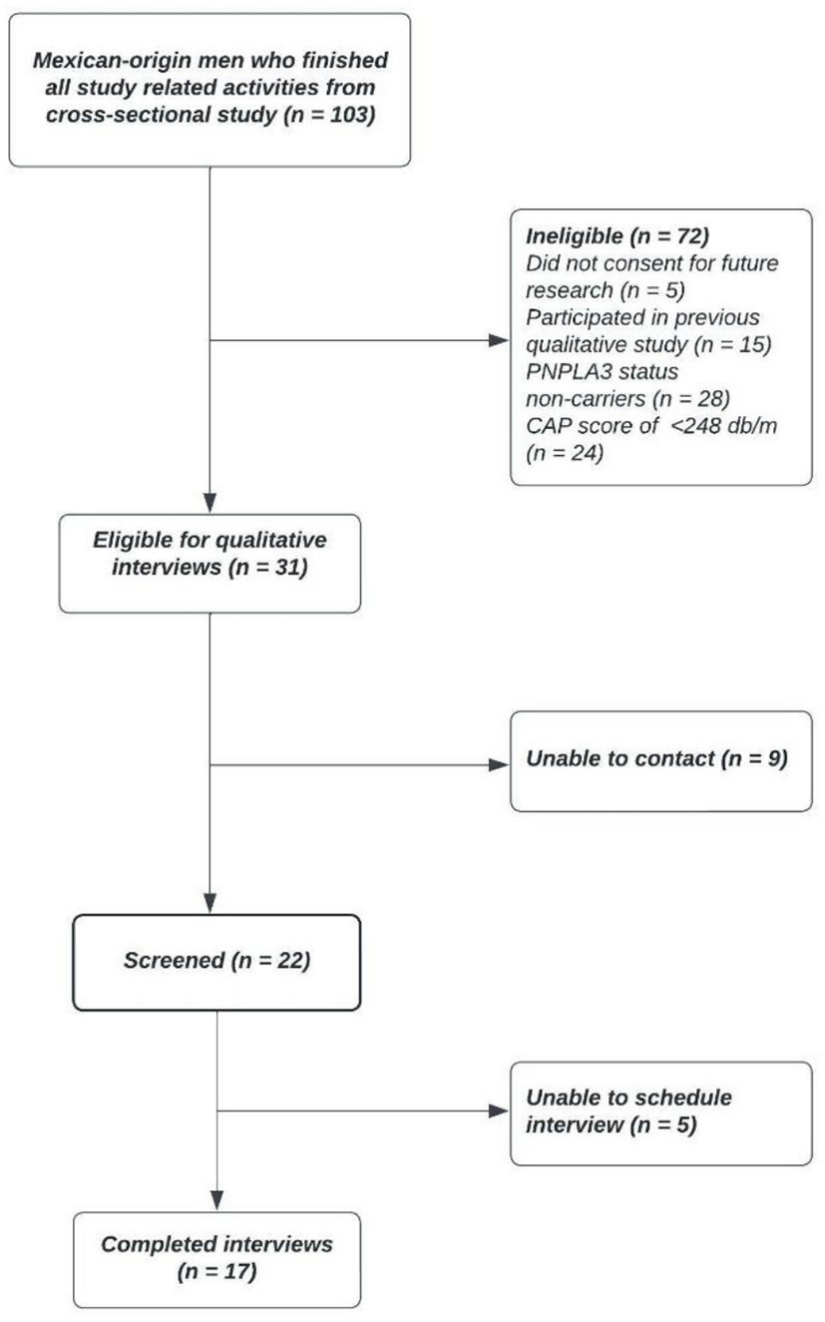
Flowchart of recruitment for in-person qualitative interviews.

Participants were recruited from a pool of eligible individuals who had given informed consent to participate in further research during a previous cross-sectional study (11). Eligible participants: (1) were carriers of *PNPLA3* (G/GG genotype); and (2) had NAFLD status identified by continuous attenuation parameter (≥248 dB/m) using a FibroScan^®^ (25), a modality of vibration-controlled transient elastography. Of the 103 prospective participants, 72 were excluded for not meeting the initial eligibility criteria (e.g., *PNPLA3* non-carriers). Out of the 31 eligible participants, research staff were unable to contact nine and unable to schedule an interview with an additional five due to time constraints, resulting in the final sample of 17 Mexican-origin men (Figure 1). All study procedures were approved by the University of Arizona Institutional Review Board (IRB# 1911187047).

### Data collection

#### Dissemination of *PNPLA3* genetic risk

Data collection occurred from September 2021 to April 2022. Genetic risk communication was disseminated following standard protocols (26). The investigative team with expertise in genetic counseling (CS), genetic risk communication (YG) and Hispanic men's health (DG, EV) developed the initial communication content. To reduce literacy demand, we considered health literacy criteria from CDC Clear Communication Index (e.g., readability, action orientation, positive tone) (27). Additionally, we developed communication content based on cultural tailoring (28). For example, we used evidential tailoring to provide evidence specific to Hispanic men based on our previous work (29). We conducted an exploratory pre-test with two community members (i.e., Mexican-origin men) and two stakeholders (i.e., community partners) and asked them to provide feedback on acceptability, appropriateness, and feasibility. The communication content was then revised and finalized based on input from community members.

Eligible and interested participants received their *PNPLA3* genetic risk status enclosed in a one-page letter mailed to their homes upon agreement of receiving such results. The use of the genetic risk letter was hypothesized to initiate disease susceptibility conversations between participants and their family members. The use of a printed letter has previously been tested and deemed optimal and preferred to disclose genetic mutations to individuals and their families (30). Two weeks after the letter was mailed, participants were contacted to ensure the letter had been received and scheduled an in-person appointment to complete the interview. The 2-week period was determined by the study to allow participants to process their genetic information and allow them to have sufficient time to share information with others if desired.

#### Semi-structured interviews

In-person semi-structured interviews were conducted in participants' preferred language (English or Spanish) by the first author (EV), a bicultural and bilingual male. This ensured a high level of cultural and gender appropriateness to maintain participant engagement and interest in the study. Data collection occurred until theoretical saturation was achieved (31). To ensure quality and precision of data collection procedures, all interviews were digitally audio-recorded and transcribed verbatim by a professional transcription service. Guided by the health belief model (HBM) (32), an interview guide was developed to gather information about Mexican-origin men's experience on receiving genetic risk results, understanding of genetic risk for NAFLD, and family communication about genetic risk (Supplemental material). Genetic counselors endorsed and contributed to the development of this interview guide to ensure questions about genetic risk were adequately prompted for participants. Upon completion of the interview, participants answered a demographics questionnaire and received a physical assessment where height, weight, and a FibroScan^®^ were completed to reassess NAFLD status. Eleven interviews (64.7%) were completed in Spanish and six (35.3%) in English. All interview sessions lasted ~30–45 min and occurred in a private room at the University of Arizona's Collaboratory for Metabolic Disease Prevention and Treatment. Participants received a $25 as a cash incentive for their time contributions to the study.

### Data analysis

Thematic analysis was used to conduct the analysis of interview transcripts (33, 34). A team-based approach was used to improve the rigor of the analytical process. Discrepancies in coding were discussed by EV, AM, and DG and revisions were made to the codebook based on the initial analysis and discussions among the coding team. Interrater agreement between the three coders was found to be adequate with a minimal percentage agreement of 80%. All final codes were sorted into themes and provided descriptive definitions and labels. To aid in the analysis process, descriptive matrix displays were created to identify code overlap and relationships among codes (35). All transcripts were analyzed in their original language to avoid misrepresentation or loss of meaning. Analyses were conducted using QSR International's NVivo qualitative data analysis software (36).

## Study findings

### Demographic characteristics

Seventeen Mexican-origin men completed the qualitative interviews. Most of the sample was identified as having the CG genotype of the *PNPLA3* risk allele (64.7%). Participants' mean age was 47.1 ± 9.1 years with an average of 22.6 ± 16.5 years living in the U.S. Mean body mass index (BMI) was 33.8 ± 6.7 kg/m^2^. Nearly half of the sample (47.1%) reported an annual household income of <$29,999 and indicated having obtained education at the level of a high school diploma/GED equivalent or less (47.0%). Most men reported having access to a primary care provider (76.5%) or health insurance coverage (70.6%). Detailed participant demographic characteristics are reported in Table 1.

**Table 1:** Participant characteristics for Mexican-origin men who completed qualitative interviews.

**Characteristics**	***n*** **/mean**	**%/range**
***PNPLA*** **3 status**		
CG	11	64.7%
GG	6	35.3%
**Language**		
Spanish	11	64.7%
English	6	35.3%
Age (years)	47.1 ± 9.1	(27–61)
Years in the US (years)	22.6 ± 16.5	(2–60)
Weight (kg)	99.6 ± 19.5	(72.2–135.7)
Body mass index (kg/m^2^)	33.8 ± 6.7	(27.0–47.5)
**Educational level**		
Grades 1–8	4	23.5%
Attended some high school	1	5.9%
Graduated high school/GED	3	17.6%
Some college	3	17.6%
Bachelor's degree	4	23.5%
Graduate degree or higher	2	11.8%
**Income (US)**		
< $29,999	8	47.1%
$30,000–$59,999	4	23.5%
>$60,000	5	29.4%
**Employed**		
Yes	15	88.2%
No	2	11.8%
**Family cancer history**		
Yes	10	58.8%
No	7	41.2%
**Access to a primary care provider**		
Yes	13	76.5%
No	4	23.5%
**Health insurance**		
Yes	12	70.6%
No	5	29.4%

### Thematic analysis

Five major themes emerged from the data including (1) assessment of delivery method; (2) understanding of genetic risk and NAFLD; (3) perceptions of genetic risk for NAFLD; (4) sharing of genetic risk information; and (5) motivators for risk reduction. We support and illustrate themes with relevant quotes from participants. Selected quotes are shown under a pseudonym to maintain participant's anonymity.

### Assessment of delivery method

Most participants (*n* = 15) gave positive feedback about the genetic risk letter that was sent to their homes. In fact, 11 men identified the genetic risk letter as their method of choice for learning this type of genetic information. Ricardo (Spanish, 59) provided the following on the didactics of the letter:

“Everything was well explained, it is in the language that I understand, and everything was very clear. It is understandable to the point.”

However, two men emphasized their preference for an in-person session after receiving the genetic risk letter. Miguel (Spanish, 44) integrated the cultural attributes of Hispanics and emphasized the importance of one-on-one interactions when obtaining genetic related information. He explained:

“…perhaps a consultation or an interview, where you explain what the results are, especially since we are talking about the Hispanic population and you know that the Hispanic population is not the type to read the entire document, and as Hispanics it makes us feel very good to have person to person contact, transmitting information face to face, especially when we have the opportunity to ask questions.”

Participants did not express strong opinions about who should communicated their genetic risk information, as long as the person was knowledgeable and capable of answering questions about how genetic risk can impact NAFLD-related outcomes. One man described:

“If you are the right person for this, go ahead, I understand, but if there is a doctor that you want to refer us to, if you send me with doctor so-and-so or to this specialist in this disease to inform us, we will also go there. Wherever you want us to go, we will go.” Rigoberto (Spanish, 52)

Further, when asked about ways to improve the dissemination of this genetic risk information, five men mentioned that having a family member present during a follow up appointment would not only facilitate their genetic risk comprehension but also create a space to receive information about NAFLD preventive measures that would benefit the family unit. Diego (Spanish, 53) stated:

“If you can have three people at the same time, then you are reaching more than one person. Like, I think giving the person the option like ‘hey, you can bring your wife, your kids, or whoever you want to talk about this.' Yeah, and if the whole family can come, that's even better.”

### Understanding of genetic risk and NAFLD

Despite participants' comprehension of their genetic predisposition for NAFLD being high (*n* = 14), a few men (*n* = 3) expressed confusion with understanding scientific terminology used in the genetic risk letter. Particularly, participants cited the expression “genetic risk” as confusing and what being carriers of the *PNPLA3* risk allele meant. Enrique (Spanish, 49) spoke about how he would have understood the concept of genetic risk better if language within the letter had included fewer medical terms. He mentioned,

“… to speak medical terms such as the so-and-so gene – many times we are not familiar with such terms, medical ones; but if it were something simpler to explain, like genes from your family or something like that, it would be easier to understand.”

Another participant suggested that the word “genetic” should be replaced by “hereditary,” which he said was more commonly used in Mexico.

“Sometimes it is a matter of word choice, instead of talking about genetic disease, they say hereditary disease and in fact in the Mexican environment… that is the most common word. It is a hereditary disease that is transmitted in the family, it is not a genetic issue.” (Miguel, Spanish, 44)

By contrast, nearly all men (*n* = 15) demonstrated modest knowledge of other genetic diseases that were described as “hereditary” or diseases that participants understood being “prone to” based on their family history. A total of 12 men recognized type 2 diabetes as a hereditary condition, eight made a reference to different types of cancer, and four mentioned other cardiovascular conditions. Antonio (English, 60) explained how cancer can run in families and shared about how his mother has been diagnosed with breast cancer.

“What could be hereditary? I guess cancer. What I understand, cancer – if somebody has it in your family, then you might get tested for it because you might have the same gene in there or something like that. My mom had breast cancer, but nobody else in the family had it.”

### Perceptions of genetic risk for NAFLD

Men were also prompted to share about their insights to receiving their genetic risk information and whether they viewed it as something positive or negative toward the way they view their health status. Most participants (*n* = 15) viewed receiving their genetic risk information as beneficial, as it generated awareness of their susceptibility to the more advanced stages of the NAFLD spectrum. Emilio (English, 27) described how learning about his genetic risk could aid him in reversing the progression of NAFLD.

“I think it's a good benefit to know. Sometimes it can be kind of scary because you don't know what you're seeing. For me, it was like ‘oh, man, something can happen to me.' I could be more likely to be with fatty liver disease, or anything like that. At the same time, being informed, I can start looking for ways to help myself.”

Of these 15 men who felt it was beneficial, 13 indicated that being made aware of their genetic risk gave them an opportunity to change their behaviors and lead a healthier lifestyle. For instance, Jose Luis (Spanish, 46) acknowledged that the process enabled him to make more conscious decisions about his physical activity and dietary habits.

“It is giving it a little bit more importance, it is giving me choice. I am aware that choices, primarily when it comes to eating habits and physical activity, can impact you at a greater to lesser degree. So with this study I am now more conscious about that.”

### Sharing of genetic risk information

It is noteworthy that all but one participant (*n* = 16) shared information about their genetic risk results with somebody from their immediate family. Thirteen men shared this information with their spouses and emphasized the importance of sharing health related information with partners. Miguel (Spanish, 44) described how cultural and gender norms played an important role in him having shared this information with his wife.

“Because you know that this matter is very cultural. The wife is the one who tells us to eat well, to not go overboard with this and that. Even when many times here in the U.S. when both of the parents work, the wife traditionally continues to be the one in charge of the kitchen.”

Additionally, three of these men shared their genetic information with at least one of their children, two shared it with at least one of their parents, and four shared it with other family members including cousins and siblings. Emilio (English, 27) explained that he shared his genetic predisposition to NAFLD with his mother because of the way her lifestyle had influenced his behaviors and both of their health statuses.

“My mom more because, obviously, I picked up my lifestyle from her…So, if it's affecting me, you know, she's older, and so it could be affecting her as well and not even knowing about it.”

Diego (Spanish, 53) provided some insights on why he decided to share this information with his children highlighting the importance of taking action and implementing lifestyle changes toward reversing the progression of NAFLD.

“I chose [my children] because we are family and at the moment I found out, I told them it is something that has to be taken care of and that there must be some changes in the way we live. Though it is not as serious right now, it can become much serious later if you don't do something to take care of yourself.”

When expanding upon participants' risk communication patterns, men were asked if they believed anyone in their families would be interested in learning about their own genetic risk for NAFLD. A total of 15 participants responded yes, suggesting a high interest in familial genetic risk testing for *PNPLA3* status among Mexican-origin adults. In fact, family members of six participants expressed immediate interest in getting genetic testing for themselves after participants shared information about their NAFLD predisposition. Emilio (English, 27) stated his mom conveyed interest in learning about her own genetic status:

“My mom felt that it was relevant to her too, because she says, ‘oh, maybe I want to get checked up as well because maybe I could have been – it could be from me going down to you, to my kids or my brothers and you'.”

### Motivators for risk reduction

Nearly all participants (*n* = 15) expressed being motivated to lower their NAFLD risk after receiving their genetic risk letter. Considering his age and high risk for developing complications of NAFLD and other chronic liver conditions, Sebastian (English, 40) highlighted that discovering his genetic predisposition motivated him to look for better approaches to take care of himself and focus on disease prevention strategies.

“Most important part is that I gotta take care of myself. I'm already, I guess, half of my life; I'm 40 years old so, it's not something that I'm worried to a point where I have to do something right away. I'm not worried, I don't feel that it's gonna affect my life, as long as I have a good lifestyle and eating habits.”

Nearly half (*n* = 7) of the men referenced their family as the main source of motivation for improving their lifestyle behaviors. Carlos (English, 38) highlighted his children as his main source of motivation for adopting strategies to help prevent NAFLD complications.

“Me, I'm a single father with three kids, no mother, my wife passed away, and my youngest is in first grade. And the liver can take you out very fast. So I don't know a lot about liver disease, I don't know a lot about it, but I know that once you get it, it goes fast. And I'm not prepared to leave my kids like that, so it's something I really gotta start looking into.”

Emilio (English, 27) also recognized the value of making lifestyle changes as an opportunity to improve his quality of life and be a part of his family's future.

“I have kids and I want to be able to see them get old. And I don't want to know that I have a higher risk of getting fatty liver disease and possibly affecting my liver so much where I'm not gonna be able to be there for them. Definitely it's something that wants to make me change my lifestyle.”

Some men described emotional responses after receiving genetic risk information and noted that these feelings served as a motivator for disease prevention through lifestyle changes. For instance, some men expressed feeling surprised (*n* = 4) and scared (*n* = 2) when first reading the letter and learning about their genetic predisposition. However, several men (*n* = 6) appeared to transition those feelings into a sense of hope when they used their genetic risk as a motivator to adopt healthier behaviors. Rigoberto (Spanish, 52) described this spectrum of emotions, when he stated,

“It is another life opportunity that is being presented to me when you told me ‘You know what? You are prone to being sick,' but if I know that I can do better at this, I am – it is a matter of how I am at the moment that I can resolve the situation, by just moving forward. That is all I have to do.”

Of note, all participants communicated being motivated and interested in participating in a prospective weight loss program focused on NAFLD health risk tailored for Mexican-origin men and provided consent to being contacted in the future for an intervention of this nature.

## Discussion

Given the influence of *PNPLA3* in the progression of liver disease and the frequency of this genetic predisposition among Mexican-origin adults (10), the current study aimed to identify appropriate strategies to communicate *PNPLA3* risk status to Mexican-origin men as a means to promote NAFLD prevention. Our study is among the first to assess Mexican-origin men's perspectives and attitudes on the delivery of genetic risk, particularly to *PNPLA3*, and the threat this conveys to the progression of the NAFLD spectrum. Participants expressed their preference in obtaining genetic risk results through a mailed letter with an optional in-person follow-up appointment with a trained health professional. In addition, almost all men were able to understand the basic implications of being carriers of the *PNPLA3* risk allele and how this affects their NAFLD risk. Overall, most participants stated that it was beneficial to learn about their genetic risk and had shared this information with at least one family member. These findings provide new evidence on how communicating *PNPLA3* risk status may help standardize the delivery of genetic testing to guide preventative measures to reduce the burden of NAFLD in this high-risk population.

Overall, men in this study supported the use of a mailed letter for communicating genetic risk, supporting prior findings for this method of delivery (30). In addition, the Spanish speaking men highlighted the importance of receiving results in their preferred language, as has been previously reported in studies centered on BRCA1/2 (14, 17). Mexican-origin men expressed their interest in having an optional appointment with either a study staff member or a health professional with sufficient knowledge on *PNPLA3* and its influence on NAFLD progression. Preference for in-person appointment is reflective of the importance of face-to-face interactions in Hispanic/Latino culture as conveyed by the construct of *personalismo* in relation to interpersonal exchanges of information (37). Parallel preferences toward face-to-face interactions were found in a study performed in rural areas in the Midwestern U.S. were Hispanic/Latino immigrants preferred obtaining clarification on health information in Spanish at in-person settings such as schools, churches, and community centers (38). As well, U.S. born Hispanic/Latinos in Cristancho et al.'s (38) cohort opted for mailed printed materials as their second preferred strategy to receive health related information. Taken together these data indicate that the initial delivery of genetic risk through a letter appears to be feasible when used with Mexican-origin men as long as consultations for further inquiries are available.

Mexican-origin men showed high levels of understanding of their genetic predisposition to NAFLD upon receiving the letter containing their *PNPLA3* risk status. In general, men demonstrated moderate awareness levels on other commonly known genetic conditions (e.g., type 2 diabetes, other cancers) but referred to them as “hereditary” rather than genetic diseases. The concept of “genetic risk” was labeled as too scientific or even confusing by participants when referring to conditions they also recognized as running in their families. Similar viewpoints were reported by Hamilton et al. (16), were Latino men and women described genetic testing as a type of blood test relating to family history or for illnesses that can be passed down from one generation to another. Independent of Hamilton's work, our sample was only composed of men from Mexican decent which allows for the development and application of culturally and gender tailored interventions and programs. Adopting gender- and culturally-specific approaches has been demonstrated to be effective in men from this population (39) and holds favorable projections with the incorporation of genetic testing (40, 41).

According to participants' testimonies, discovering their *PNPLA3* status allowed them to assess their risk for liver diseases and the implications to more severe stages of disease in the NAFLD continuum. As derived from the HBM, participants in our study acknowledged that their newfound perceptions of NAFLD risk may influence the adoption of disease prevention strategies such as changes in diet and physical activity. Most participants of this study considered learning about their genetic risk status as a definite benefit to their health given the potential improvement, they can achieve by implementing such lifestyle changes. Similar actionable health information were observed in Hamilton et al. (16), in which both, Latino men and women anticipated genetic risk as a source of additional information for them to protect their health and their children's through the engagement of new healthier behaviors.

Men in our study had comparably familial views as they believed sharing their genetic risk information with family members would allow their entire family to become aware of the disease and come together and partake in lifestyle modification efforts. This finding relates closely to the cultural value of *familismo*, a core component of Latino/Hispanic individuals characterized as the strong interconnectedness and attachment with nuclear and extended family members (42, 43). All but one participant testified to have shared information about their genetic risk predisposition to at least one close family member. Though this was an expected finding, we found that the propagation of participants' genetic risk generated interest in family members indicating that genetic testing in Mexican-origin adults might be more viable than anticipated. This finding along with the presence of *familismo* could help inform the utilization of family-based approaches for the use and delivery of genetic testing to communicate NAFLD risk and disease reduction strategies for Mexican-origin families.

Findings from this sample of Mexican-origin men suggest a clear preference for learning about the role genetics plays in determining NAFLD risk. They expressed high levels of motivation to reduce their NAFLD progression with main motivators identified as improving their health status and being present in their children's life for much longer. Similar conclusions were seen in a previous qualitative study where familial and cultural attitudes appeared to enhance perceived risk for NAFLD progression in Mexican-origin men (44).

Lastly, it is important to consider emotional vulnerability when delivering genetic testing results to inform disease risk, and how emotions may motivate or discourage positive change. Limited evidence has shown the relevance of emotional screening in this population, but has particularly focused on women (45). A few men in our study reported initially feeling surprised and afraid of the news of being genetically predisposed to NAFLD, and using those feelings as an opportunity to gain self-confidence tied to their ability to change their health status and reduce their odds of progressing to more serious stages of liver disease. Similar emotional responses were evidenced in another sample of Mexican-origin men and women from our initial recruitment cohort in which participants were interviewed to measure NAFLD awareness, knowledge and perceptions of disease after receiving their results from a FibroScan^®^ (46). Consistent with information processing theories (47), credible health risk information can activate emotional responses such as distress and fear. Taking opportunities to encourage pro-health behaviors to lower distress could results in greater uptake of recommended actions (e.g., lifestyle changes).

### Strengths and limitations

This qualitative study presents several strengths. This is the first known study to communicate *PNPLA3* risk status to Mexican-origin men. The process of genetic risk communication is critical considering the high and growing burden of NAFLD in this subpopulation along with the forthcoming rise of utilizing genetic testing as an opportunity to promote disease prevention. Another asset of our study design was having an interdisciplinary approach, including community members and stakeholders, guiding the development of the genetic risk letter, adding a further degree of value to the study's validity. In addition, our qualitative study design offers a nuanced exploration of the lived experiences of Mexican-origin men. However, the study has several limitations, including the variations in time between when participants become aware of their genetic predisposition through the letter and the time, they attended interview sessions. Due to availability conflicts, some men (*n* = 4) were unable to schedule their interview time by the two-week mark determined by the study and attended their interview up to 3 months after receiving the letter. This could have impacted participants' retention of information and cause recall bias surrounding the experiences they had lived since becoming aware of their genetic risk. Another limitation relates to men's previous exposure to NAFLD knowledge because of their participation in the initial cross-sectional study. This previous involvement would also consider all 17 participating men as part of the highest risk group to NAFLD due to the implications of the initial eligibility criteria from such study (e.g., body mass index > 25.0 kg/m^2^). Lastly, it is important to acknowledge cost considerations related to genetic testing as a barrier for the general population to obtain this kind of testing. Particularly, *PNPLA3* genetic testing is not publicly available and may hinder efforts to widely scale result dissemination (48). Future studies should consider the evaluation of delivering *PNPLA3* risk status to low-risk individuals considered at lower risk, other Hispanic subpopulations, and women to compare views on risk susceptibility and severity.

## Conclusions

This qualitative analysis provides preliminary outcomes on the benefit of integrating genetic risk communication of *PNPLA3* status as a component of disease prevention in a high-risk subpopulation. The overall experiences of these men indicate high levels of interest and feasibility in the delivery of genetic risk status to Mexican-origin men. The development of relevant family-based interventions that are culturally tailored for this high-risk group warrants further research to evaluate its effectiveness in decreasing the burden of NAFLD and overall liver cancer mortality.

## Data availability statement

The datasets presented in this article are not readily available because data is protected under the National Institutes of Health's Certificate of Confidentiality to protect participants confidentiality. Requests to access the datasets should be directed at: davidogarcia@arizona.edu.

## Ethics statement

The studies involving human participants were reviewed and approved by University of Arizona Institutional Review Board (IRB# 1911187047). The patients/participants provided their written informed consent to participate in this study.

## Author contributions

DG, EV, RC, YG, and CS designed the research. EV and DG conducted the research. EV, DG, and AM analyzed data. All authors were involved in writing the paper and had final approval of the submitted and published versions.
