# Supplementary material for: Communicating PNPLA3 genetic risk status for NAFLD among Mexican-origin men
Source: Front Public Health. 2023 Jan 4;10:1090101. doi: 10.3389/fpubh.2022.1090101 (PMC9846364; doi:10.3389/fpubh.2022.1090101)
Supplement: Supplementary file 1 [file Data_Sheet_1.docx]

| **Introductions**  Hello, my name is [first name]. Thank you for being here with me today. I have us scheduled for 60-90 minutes together. Does that still work for you? I want to make sure we honor our time constraints today. In addition, I want to remind you that your participation in this interview is voluntary, and you may stop at any time you wish. At the end of this interview, you will be compensated $25 for your time.  Our goal for these interviews is to learn more about what Mexican-origin men know about non-alcoholic fatty liver disease (NAFLD) health risk, specifically genetic risk, and how that may influence how people work to manage their body weight.  When we start the interview, I would like to begin voice recording our conversation. I will also be taking some notes as we go, but the tape recording will ensure that I have a complete record of our conversation. Your thoughts and experiences are important to us, so we want to do our best to capture your words accurately. Do I have your permission to record our conversation today?  I would like to remind you that everything that you say will be confidential. All recordings will be securely stored here in our research office (Collaboratory for Metabolic Disease Prevention and Treatment). Recordings will be transcribed, and original recordings will be destroyed. The research team will summarize this interview and use your contribution to develop results and conclusions for the research, but any information that can identify you will be removed.  I will now begin recording. [Begin recording]  _____________________________________________________________________________ |
| --- |
|  |

**Part 1: Knowledge and Considerations of Genetic Risk**

“I’d like to begin today by discussing with you the idea of genetic risk of NAFLD. This discussion is related to the results of the letter that we mailed to you 2-4 weeks ago.”

**Experience of getting genetic risk results**

- Do you remember the moment you received the letter and first read your genetic risk results?
  - What went through your mind at that time? How did you respond?
- What do you think are the benefits of getting this risk information?
- Do you think there are any negative sides to learning about your genetic risk?
- Do you think your genetic risk for NAFLD means that other members of your family share that risk? How does that make you feel?
- Did you find the letter clear and easy to understand or not? If not, what would have made it easier to understand?
  - Is there a different way (other than a letter) that you would rather have received your genetic risk information?

**Understanding of genetic risk of NAFLD**

- What do you think was the most important thing you learned from the genetic risk letter we sent you?
- Thinking back to what you read in the letter, can you describe the *PNPLA3* gene in your own words?
  - What else do you know about genetic risk factors for other diseases?
- What other risk factors do you know about that are not genetic for NAFLD?
- Did getting your genetic risk influence how you think about your own health?

**Family communication about genetic risk for NAFLD**

- Have you shared your genetic risk with your family members? Why or why not?

*If yes…*

- - Who did you share your risk information with? Why did you choose that person/people?
    - What was it like talking to your family member(s) about your genetic risk? How did it go? What did you talk about?
    - What was their reaction? How did their reaction make you feel?
    - Did they feel that the information was relevant to their own health or only about you and your health?
    - Does anyone in your family want to learn about their own genetic risk?
      - If so, do you think having someone there to help would have made talking to your family about genetic risk easier? If yes, who would be the best person to help with that?
      - Is there any other support that out team can provide?

*If no…*

- - Why did you decide not to share your genetic risk information with your family?
    - Is there something that worries you or concerns you about talking about it? Do you not think it is important to share the information?
  - Do you think having someone there to help would have made talking to your family about genetic risk easier? If yes, who would be the best person to help with that?
  - Is there any other support that out team can provide?

**Part 2: Closing**

“We will now wrap up this session with a few more questions.”

**Perceptions regarding the addition of genetic risk to a weight loss program:**

- Does learning that you have elevated genetic risk for NAFLD change the way you view the importance of weight loss?
- Do you think that now that you know about your elevated genetic risk for NAFLD you are more motivated to try harder and be successful in a weight loss program? Why? Why not?
- Aside from what we have spoken about today, is there any way that our program can support you or assist you in working to reduce your NAFLD risk?
- Would you be interested in participating in a weight loss program focused on NAFLD health risk for Mexican-origin men? Do we have your permission to contact you once we are ready to recruit?

Thank you for your time today and for all the information you have shared with us. This is the end of the interview, and we will now pay you for your time.

**Introducción**

Hola, mi nombre es [nombre]. Gracias por estar aquí hoy. Tenemos programados aproximadamente 60 a 90 minutos para la entrevista de hoy. ¿Eso todavía funciona para usted? Quiero asegurarme de que respetamos nuestro tiempo asignado. Además, me gustaría recordarle que su participación en esta entrevista es voluntaria y puede detenerse en cualquier momento que usted desee. Al final de esta entrevista, se le compensará $25.00 por su tiempo.

Nuestro objetivo con estas entrevistas es aprender más sobre lo que los hombres de origen mexicano saben sobre el riesgo de la enfermedad del hígado graso no alcohólico (NAFLD por sus siglas en ingles), específicamente sobre el riesgo genético y como eso pueda influir en la manera en que las personas trabajen para manejar su peso.

Cuando comencemos la entrevista, me gustaría grabar nuestra conversación. También estaré tomando algunas notas a medida que avancemos, pero la grabación de voz asegurará que no se me pase ningún detalle de nuestra conversación. Sus pensamientos y experiencias son realmente importantes para nosotros, por lo que queremos hacer nuestro mayor esfuerzo para capturar sus palabras con precisión. ¿Tengo su permiso para grabar nuestra conversación el día de hoy?

Me gustaría recordarle que todo lo que usted diga será confidencial. La grabación se almacenará de forma segura en nuestras oficinas de investigación (Centro de Colaboración para la Prevención y el Tratamiento de Enfermedades Metabólicas). Grabaciones serán transcritas y las grabaciones originales serán destruidas. El equipo de investigación resumirá esta entrevista y utilizará su contribución para desarrollar resultados y conclusiones para la investigación, pero se eliminará toda información que pueda identificarlo a usted.

En este momento comenzare a grabar. [Comience a grabar]

______________________________________________________________________________

**Parte 1: Conocimiento y Consideraciones sobre el Riesgo Genético**

“Me gustaría comenzar a platicar con usted sobre la idea del riego genético para el hígado graso. Este tema está relacionado a la carta que le mandamos hace unas 2-4 semanas con sus resultados.

**Experiencia al recibir resultados sobre el riesgo genético**

- ¿Usted recuerda el momento en que recibió su carta y leyó sus resultados sobre su riesgo genético?
  - ¿Qué paso por su mente en ese momento? ¿Cómo reacciono?
- ¿Cuáles son los beneficios que usted ve al recibir esta información sobre su riesgo?
- ¿Usted cree que hay algún lado negativo al aprender sobre su riego genético para esta enfermedad?
- ¿Usted cree que su riesgo genético al hígado graso significa que otros miembros de su familia también compartan ese riesgo? ¿Cómo lo hace sentir eso?
- ¿Pensó que la carta fue fácil de entender? Si no, ¿qué hubiera ayudado a que fuera mas fácil de comprender?
  - ¿Hay algún otro método (además de la carta) que usted hubiera preferido para recibir su información sobre su riesgo genético?

**Comprendiendo el riesgo genético para el hígado graso**

- ¿Qué piensa que fue lo más importante que usted aprendió de la carta sobre su riesgo que le mandamos?
- Recordando lo que leyó en su carta, ¿podría describirme lo que el gen *PNPLA3* es en sus propias palabras?
  - ¿Qué más sabe usted sobre genes de riesgo para otras enfermedades?
- ¿Qué otros factores de riesgo usted conoce sobre el hígado graso (excluyendo la genética)?
- ¿Usted cree que el recibir su riesgo genético ha influido en cómo percibe so salud?

**Comunicación familiar sobre el riesgo genético al hígado graso**

- ¿Ha compartido la información sobre su riesgo genético a miembro de su familia? ¿Por qué o porque no?

*Si respondió si…*

- - ¿Con quien compartió la información sobre su riesgo? ¿Por qué eligió a esa persona/s?
    - ¿Puede contarme como se dio esa conversación sobre su riesgo genético? ¿Cómo fue? ¿De qué se habló, que otros temas fueron hablados?
    - ¿Cuál fue la reacción de sus familiares? ¿Cómo lo hizo sentir su reacción?
    - ¿Acaso ellos/as sintieron que la información fue relevante a su propia salud, o solo sobre usted y su salud?
    - ¿Habrá alguien de su familia que quisiera aprender sobre su propio riesgo genético?
      - Si es así, ¿usted piensa que el tener a alguien ahí para ayudarlo hubiera hecho más fácil el comunicar su riesgo genético con su familia? Si es así, ¿Quién seria la mejor persona para ayudarlo con eso?
      - ¿Hay algún otro tipo de apoyo que nuestro equipo pudiera ayudarlo?

*Si respondió no…*

- - ¿Por qué decidió no compartir la información de su riesgo genético con algún familiar?
    - ¿Acaso hay algo que lo preocupa o concierna al hablar sobre su riesgo genético?
  - ¿Usted piensa que el tener a alguien ahí para ayudarlo hubiera hecho más fácil el comunicar su riesgo genético con su familia? Si es así, ¿Quién sería la mejor persona para ayudarlo con eso?
  - ¿Hay algún otro tipo de apoyo que nuestro equipo pudiera ayudarlo?

**Parte 2: Cierre**

“Muy bien, estamos a punto de terminar con esta sesión, con solo unas preguntas finales.”

**Percepciones sobre la integración del riesgo genético a un programa de pérdida de peso:**

- ¿Acaso cambio la forma en que usted ve la importancia de la pérdida de peso ahora que sabe que tiene un riesgo genético elevado al hígado graso?
- ¿Usted cree estar más motivado a dar un esfuerzo mayor y ser exitoso en un programa de perdida de paso ahora que sabe sobre su riesgo genético al hígado graso? ¿Por qué o por qué no?
- Aparte de todo lo que platicamos el día de hoy, ¿hay algo en lo que nuestro programa lo pueda ayudar o apoyar en sus esfuerzos para reducir su riesgo al hígado graso?
- ¿Le interesaría participar en un programa de pérdida de peso enfocado en el riesgo y prevención del hígado graso para mexicanos de origen mexicano? ¿Nos da permiso para contactarlo ya que estemos listos para reclutar participantes?

Muchas gracias por su tiempo el día de hoy y por la información que nos acaba de compartir. Este es el fin de la entrevista y ahora le pagaremos por su tiempo.
